# Surgery for metastatic tumors of the pancreas

**DOI:** 10.1186/s40792-017-0308-0

**Published:** 2017-02-18

**Authors:** Taisuke Yagi, Daisuke Hashimoto, Katsunobu Taki, Kensuke Yamamura, Akira Chikamoto, Masaki Ohmuraya, Toru Beppu, Hideo Baba

**Affiliations:** 1 Department of Gastroenterological Surgery, Kumamoto University Graduate School of Medical Sciences, 1-1-1 Honjo, Kumamoto City, 860-8556 Japan; 2 Department of Gastroenterological Surgery, Institute of Resource Development and Analysis, Kumamoto University Graduate School of Medical Sciences, Kumamoto City, Japan

**Keywords:** Metastasis, Pancreas, Operation

## Abstract

Metastatic lesion of the pancreas originated from other organs is uncommon. The aim of this report was to evaluate the outcome of surgery in patients with isolated metastases to the pancreas. Nine patients underwent pancreatic resection for metastatic malignant disease from 2000 to 2015 at the Department of Gastroenterological Surgery of the Kumamoto University Hospital. The primary lesion was renal cell carcinoma in 7 cases, colon cancer in 1 and malignant melanoma in 1. The median interval from the initial operation to pancreatic resection was 138 months. Operative procedure was distal pancreatectomy in 6 cases, pancreaticoduodenectomy in 2 and total pancreatectomy in 1. Two patients with renal cell carcinoma and 1 patient with malignant melanoma died 131, 108, and 4 months after the pancreatic resection, respectively. Other 6 patients have survived until now with 23.5 months of observation periods after pancreatic resection. In conclusion, pancreatic metastasis can develop years after the treatment of primary lesion. Pancreatic resection can achieved long-term survival, at least in the patients who had primary renal carcinoma.

## Background

Most pancreatic tumors are primary pancreatic adenocarcinoma. However, metastatic pancreatic tumor can be developed from renal cell cancer, lung, breast, colon, or skin tumors [1–7]. Metastasis to the pancreas is rare, accounts for less than 2% of all pancreatic malignancies [3, 4, 8–11] and can be generally developed synchronous or metachronous and single or multiple. In addition, a previous large autopsy series indicated that the prevalence of pancreatic metastases was 6 to 11%, and renal cell carcinoma was the most common primary tumor to cause metastatic pancreatic tumors [12].

Experience with resections of pancreas for the isolated metastatic lesions is very limited [3, 6–8, 10, 11, 13, 14], because metastatic disease to the pancreas is considered to exist commonly with metastasis to other organs such as the liver and lung [1]. As a result, the value of surgical treatment to the metastasis to the pancreas is unclear, and there are no guidelines or recommended strategies regarding the appropriate management of such lesions.

The aim of this report was to evaluate the outcome of surgery in patients with metastases to the pancreas.

## Case presentation

### Patients’ characteristics, primary tumors, and other metastasis before pancreatic metastasis

Nine patients underwent pancreatic resection for metastatic malignant disease from 2000 to 2015 at the Department of Gastroenterological Surgery of the Kumamoto University Hospital. The patients included 5 males and 4 females, with a median age of 66 years (range, 52–83) at the pancreatic surgery (Table 1). The primary lesion, clear cell renal cell carcinoma (RCC) (right kidney in 3, left in 3, and bilateral in 1), 1 rectal cancer (tubular adenocarcinoma), and 1 oral malignant melanoma (MM), was resected in all cases.

**Table 1:** Patients’ characteristics, primary tumors, and other metastasis before pancreatic metastasis

Case no.	Age at the pancreatic surgery/gender	Primary tumors			Other metastasis before pancreatic metastasis	
		Location	Treatment	Histology	Location	Treatment
1	61/F	Left kidney	Resection	Clear cell RCC	–	–
2	52/M	Bilateral kidney	Resection	Clear cell RCC	–	–
3	67/F	Left kidney	Resection	Clear cell RCC	–	–
4	83/M	Right kidney	Resection	Clear cell RCC	–	–
5	66/M	Left kidney	Resection	Clear cell RCC	Bilateral lung	Axitinib → Sunitinib
6	69/F	Right kidney	Resection	Clear cell RCC	–	–
7	55/F	Right kidney	Resection	Clear cell RCC		
8	72/M	Rectum	Resection	Tubular adenocarcinoma	Right lung	Resection
9	55/M	Oral cavity	Resection	Malignant melanoma	–	–

*RCC* renal cell carcinoma

Before the emergence of the pancreatic metastasis, 2 patients experienced metastasis of the other organs (Table 1). Bilateral multiple lung metastasis from RCC was developed in case no. 5, 10 months before the pancreatic metastasis. It was treated by axitinib, followed by sunitinib, and archived partial response (PR). In this case, the lung metastases were well-controlled by chemotherapy. However, pancreatic metastasis was growing, and so distal pancreatectomy was performed. A solitary right lung metastasis from rectal cancer in case no. 7 was resected 76 months before the pancreatic metastasis.

### Characteristics of the pancreatic metastasis and results of the pancreatic surgery

**Fig. 1:**
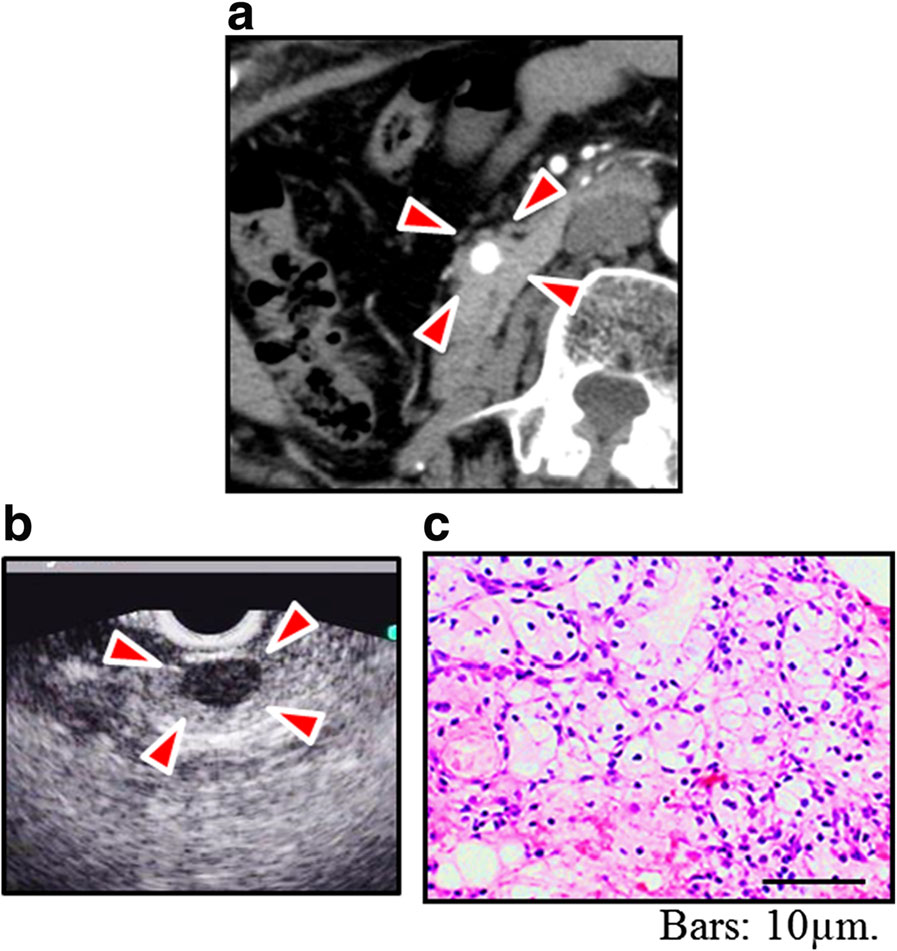
Preoperative findings of case no. 6. Enhanced CT (**a**) and EUS (**b**) revealed 10-mm tumor (*arrowheads*) in the pancreatic head of case no. 6. It was preoperatively diagnosed as metastasis from clear cell RCC (**c**) by EUS-FNA

The median interval from the initial surgery to the emergence of the pancreatic metastasis of the whole cases was 138 months (range, 0–228). The interval was 138 months (range, 0–228) in RCC patients, 154 months in a rectal cancer patient, and 5 months in a MM patient, respectively (Table 2). Pancreatic metastasis was solitary in 6 cases and multiple in 3 cases and existed in the head in 2 cases, in the body–tail in 6 cases, and in the whole pancreas in 1 (case no. 7). Interestingly, metastasis from the left kidney was developed in pancreatic body–tail in all cases (nos. 1, 3, and 5). The median size of the largest pancreatic metastasis was 28 mm (range, 10–39). In case no. 6, whereas the pancreatic tumor was only 10 mm (Fig. 1a), it could be preoperatively diagnosed as metastasis from clear cell RCC by endoscopic ultrasound-guided fine needle aspiration (EUS-FNA) (Fig. 1b, c).

**Table 2:** Characteristics of the pancreatic metastasis and results of the pancreatic surgery

Case no.	Interval (months)	Location	Tumor number	Size (mm)	Operative procedure	Operative time (min)	Operative bleeding (g)	Postoperative complication	Histology
1	60	Tail	2	14, 25	DP	241	467	None	Clear cell RCC
2	0	Head	1	36	PPPD	448	710	DGE	Clear cell RCC
3	138	Body–tail	3	17, 22, 28	DP	299	580	None	Clear cell RCC
4	156	Body–tail	1	30	DP	328	136	None	Clear cell RCC
5	228	Tail	1	39	DP	440	2587	None	Clear cell RCC
6	144	Head	1	10	SSPPD	472	745	POPF	Clear cell RCC
7	26	Head–body–tail	6	6~20	TP	406	1176	None	Clear cell RCC
8	154	Tail	1	35	DP	324	445	POPF	Tubular adenocarcinoma
9	5	Body–tail	1	28	DP	263	356	None	Malignant melanoma

*Interval* months from the initial surgery to the operation for the pancreatic metastasis, *DGE* delayed gastric emptying, *DP* distal pancreatectomy, *POPF* postoperative pancreatic fistula, *PPPD* pylorus-preserving pancreaticoduodenectomy, *RCC* renal cell carcinoma, *SSPPD* subtotal stomach-preserving pancreaticoduodenectomy, *TP* total pancreatectomy

**Fig. 2:**
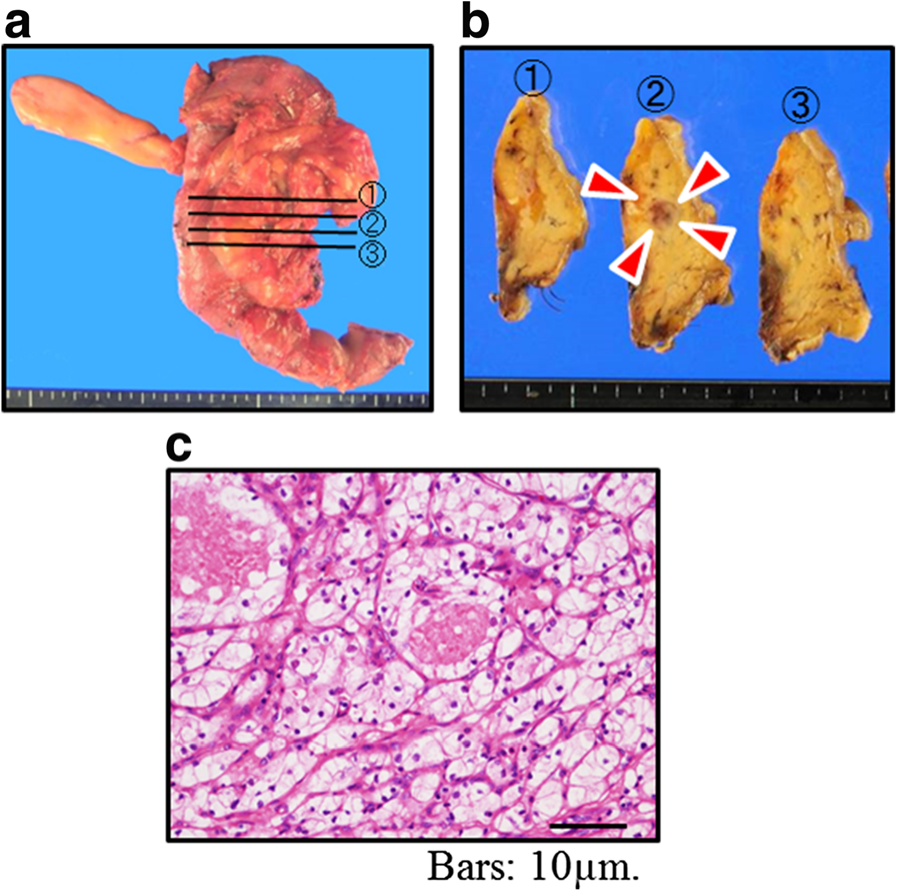
Operative and histological findings of case no. 6. SSPPD (**a**) was performed in case no. 6. Histologically, the tumor (*arrowheads*) (**b**) was confirmed as the metastasis from clear cell RCC (**c**)

Operative procedure was distal pancreatectomy (DP) in 6 cases, pancreaticoduodenectomy (PD) in 2 cases (pylorus-preserving pancreaticoduodenectomy (PPPD) in 1 and subtotal stomach-preserving pancreaticoduodenectomy (SSPPD) in 1), and total pancreatectomy (TP) in 1 case (Table 2, Fig. 2a, b). Median operative time was 328 min (range, 241–472), and median operative blood loss was 580 g (range, 136–2587). Postoperative complication was observed in 3 (33.3%), grade B of postoperative pancreatic fistula (POPF) in 2, and delayed gastric emptying (DGE) in 1. There was no hospital death. Pathological diagnoses of the pancreatic tumors were consistent with those of the primary lesion in all cases (Fig. 2c).

### Other metastasis after pancreatic metastasis and long-term outcome

Bilateral multiple lung metastasis existed in 3 patients with metastasis from RCC, and 2 cases of them were treated by combination of interferon (IFN), interleukin-2 (IL-2), and tegafur-uracil (UFT) or sunitinib, respectively (Table 3). Bilateral multiple lung metastasis and bone metastasis developed in a patient with metastasis from MM, treated by dacarbazine.

**Table 3:** Other metastasis after pancreatic metastasis and long-term outcome

Case no.	Recurrence or metastasis after pancreatic resection		Postoperative observation period (months)
	Location	Treatment	
1	Bilateral lung	None (BSC)	131^a^
2	Bilateral lung	IFN + IL-2 + UFT	108^a^
3	–	–	138
4	–	–	49
5	Bilateral lung	Sunitinib	8
6	–	–	8
7	–	–	3
8	–	–	39
9	Bilateral lung, bone	Dacarbazine	4^a^

*Postoperative observation period* months after the operation for the pancreatic metastasis, *BSC* best supportive care, *IFN* interferon, *IL-2* interleukin-2, *UFT* tegafur-uracil

^a^Dead

Two patients with metastasis from RCC and 1 patient with metastasis from MM died 131, 108, and 4 months after the pancreatic resection, respectively. Other 6 patients have survived until now with 23.5 months (range, 3–138) of observation periods after pancreatic resection.

## Conclusions

The pancreas is an unusual but occasionally favored site for metastasis, notably from carcinoma of the kidney and lung [1–3, 15]. In this series of the patients, 77.8% (case nos. 1–7) pancreatic tumor were metastasis from clear cell RCC and 11.1% (case no. 7) were from rectal cancer, consistent with previous studies [4–7]. Interestingly, all metastatic tumors from the left RCC (3 cases) were developed in the left side of the pancreas. This tendency of metastatic direction to the pancreas has been never reported and may indicate the mechanism of hematogenous metastasis from the kidney to pancreas. In patients with portal hypertension, vascular endothelial growth factor (VEGF)-dependent angiogenesis plays a crucial role in the formation of portal-systemic collateral vessels, which include spleno-renal shunts [16]. VEGF has an important role in progression of RCC [17]. Collateral vessels like spleno-renal shunts dependent on VEGF angiogenesis possibly contribute to the tendency of metastatic direction in RCC patients. One case of our series was the metastasis from oral malignant melanoma, with poor prognosis even the aggressive chemotherapy by dacarbazine. There were a few reports indicated similar situation [18, 19]; however, it appears that surgical resection is only a palliative procedure, because long-term survival is a rare event.

Despite the technological advances, preoperative diagnosis of the metastatic pancreatic tumor is sometimes difficult [15]. EUS-FNA is an excellent method for procurement of diagnostic samples from the pancreas, with a diagnostic accuracy of more than 90% for pancreatic adenocarcinoma [20, 21]. One patient in this study (case no. 6) was preoperatively diagnosed as metastasis of clear cell RCC by EUS-FNA, even only 10 mm of the size of the tumor. It suggested that EUS-FNA is useful to correct diagnosis of metastatic pancreatic tumor.

Surgical resection of the metastatic tumor to the pancreas should be approached by carefully applying appropriate selection criteria, because of the substantial morbidity indicated in this series and a previous report [22]. On the other hand, a noteworthy finding in our series was a long interval from the initial surgery to the pancreatic metastasis and long survival after pancreatic resection. This was particularly the cases with clear cell RCC patients. Yuasa et al. reported that the median duration from diagnosis of RCC to pancreatic metastasis was 7.8 years (4.2–12.7 years) [23]. Consistent with the study, metastatic tumor of the pancreas from RCC is recurred in long-term interval in general in our series. Patients with isolated RCC metastasis to the pancreas, whether synchronous or metachronous, represent a selected group of patients with more indolent RCC [22]. In addition, new effective therapeutic strategy such as molecular target drug has been introduced to metastatic disease from clear cell RCC [24]. This advantage may provide the benefit of resection of the pancreatic metastasis even with metastatic lesions in other organs such as the lung, in selected cases with good response to the treatment, as indicated in our study.

We performed distal pancreatectomy in 6 cases, pancreaticoduodenectomy in 2 cases, and total pancreatectomy in 1 case. The surgical strategy for pancreatic metastatic tumor has not been established. Recently, limited or partial pancreatectomy has been performed, especially for disease which does not need lymph node dissection [25]. These preserving operations may be useful for patients with pancreatic metastasis from clear cell renal cell carcinoma.

In conclusion, the metastasis to the pancreas can be developed years after the initial surgery of the primary disease. EUS-FNA was suggested to be feasible for diagnosis and helpful to consider the indication of surgery. Long-term survival can be archived with pancreatic resection, especially in patients with pancreatic metastasis from clear cell RCC.

## Abbreviations

DGE: Delayed gastric emptying
DP: Distal pancreatectomy
EUS-FNA: Endoscopic ultrasound-guided fine needle aspiration
IFN: Interferon
IL-2: Interleukin-2
MM: Malignant melanoma
PD: Pancreaticoduodenectomy
POPF: Postoperative pancreatic fistula
PPPD: Pylorus-preserving pancreaticoduodenectomy
PR: Partial response
RCC: Renal cell carcinoma
SSPPD: Subtotal stomach-preserving pancreaticoduodenectomy
TP: Total pancreatectomy
UFT: Tegafur-uracil
